# Use of genome sequencing to hunt for cryptic second-hit variants: analysis of 31 cases recruited to the 100 000 Genomes Project

**DOI:** 10.1136/jmg-2023-109362

**Published:** 2023-08-09

**Authors:** A Rachel Moore, Jing Yu, Yang Pei, Emily W Y Cheng, Ana Lisa Taylor Tavares, Woolf T Walker, N Simon Thomas, Arveen Kamath, Rita Ibitoye, Dragana Josifova, Anna Wilsdon, Alison Ross, Alistair D Calder, Amaka C Offiah, Andrew O M Wilkie, Genomics England Research Consortium, Jenny C Taylor, Alistair T Pagnamenta

**Affiliations:** 1 Wellcome Centre for Human Genetics, NIHR Oxford Biomedical Research Centre, University of Oxford, Oxford, UK; 2 Cambridge Genomics Laboratory, Cambridge University Hospitals NHS Foundation Trust, Cambridge, UK; 3 Nuffield Department of Clinical Neurosciences, University of Oxford, Oxford, UK; 4 Clinical Genetics Group, MRC Weatherall Institute of Molecular Medicine, University of Oxford, Oxford, UK; 5 Genomics England Limited, London, UK; 6 School of Clinical and Experimental Sciences, Faculty of Medicine, University of Southampton, Southampton, UK; 7 PCD Centre, University Hospital Southampton NHS Foundation Trust, Southampton, UK; 8 Wessex Regional Genetics Laboratory, Salisbury NHS Foundation Trust, Salisbury, UK; 9 All Wales Medical Genomics Service, University Hospital of Wales, Cardiff, UK; 10 North West Thames Regional Genetics Service, Northwick Park Hospital, Harrow, London, UK; 11 Department of Clinical Genetics, Guy's and St Thomas' Hospitals NHS Trust, London, UK; 12 Clinical Genetics, Nottingham City Hospital, Nottingham, UK; 13 Clinical Genetics, NHS Grampian, Aberdeen, UK; 14 Radiology Department, Great Ormond Street Hospital for Children NHS Foundation Trust, London, UK; 15 Department of Oncology and Metabolism, The University of Sheffield, Sheffield, UK

**Keywords:** diagnosis, genetic diseases, inborn, genetics, medical, sequence analysis, DNA, genomics

## Abstract

**Background:**

Current clinical testing methods used to uncover the genetic basis of rare disease have inherent limitations, which can lead to causative pathogenic variants being missed. Within the rare disease arm of the 100 000 Genomes Project (100kGP), families were recruited under the clinical indication ‘single autosomal recessive mutation in rare disease’. These participants presented with strong clinical suspicion for a specific autosomal recessive disorder, but only one suspected pathogenic variant had been identified through standard-of-care testing. Whole genome sequencing (WGS) aimed to identify cryptic ‘second-hit’ variants.

**Methods:**

To investigate the 31 families with available data that remained unsolved following formal review within the 100kGP, SVRare was used to aggregate structural variants present in <1% of 100kGP participants. Small variants were assessed using population allele frequency data and SpliceAI. Literature searches and publicly available online tools were used for further annotation of pathogenicity.

**Results:**

Using these strategies, 8/31 cases were solved, increasing the overall diagnostic yield of this cohort from 10/41 (24.4%) to 18/41 (43.9%). Exemplar cases include a patient with cystic fibrosis harbouring a novel exonic LINE1 insertion in *CFTR* and a patient with generalised arterial calcification of infancy with complex interlinked duplications involving exons 2–6 of *ENPP1*. Although ambiguous by short-read WGS, the *ENPP1* variant structure was resolved using optical genome mapping and RNA analysis.

**Conclusion:**

Systematic examination of cryptic variants across a multi-disease cohort successfully identifies additional pathogenic variants. WGS data analysis in autosomal recessive rare disease should consider complex structural and small intronic variants as potentially pathogenic second hits.

**WHAT IS ALREADY KNOWN ON THIS TOPIC**

- Although it is known that whole genome sequencing can uncover cryptic structural variants (SVs) and deep intronic splice variants, most ‘second-hit’ studies in the current literature are limited to specific genes or disease areas.

**WHAT THIS STUDY ADDS**

- By assessing a clinically heterogeneous cohort from the 100k Genomes Project (100kGP), we highlight the diagnostic uplift that can be achieved by systematically assessing SVs in combination with the use of *in silico* splice prediction tools.
- Our results also demonstrate that the mutational spectra of variants in *CFTR* can include partial LINE1 insertions and strengthen the case for c.3874–4522A>G to be included in variant panel testing.

**HOW THIS STUDY MIGHT AFFECT RESEARCH, PRACTICE OR POLICY**

- The study helps to highlight the importance of close interaction between data analysts and clinicians.
- Cryptic pathogenic structural and splice variants often remain undetected in the 100kGP.
- Optical genome mapping and RT-PCR can be effective methods for resolving complex SVs that remain ambiguous with short-read genome sequencing data.

## Introduction

Complex or cryptic variants are an area of ongoing interest and discovery in inherited disease. However, clinical diagnostics technologies, testing strategies and bioinformatic pipelines have certain limitations, which may mean some pathogenic variants are not detected. Common clinical techniques such as SNP array have allowed the discovery of microdeletion and microduplication syndromes^1^; recurrent small structural variants (SVs) have also been reported, for example, multi-exon deletions in *CFTR*.^2^ However, the limited resolution of arrays, along with their inability to detect balanced SVs^3^ and the difficulty of interpreting complex variants, can hinder detection of pathogenic variants.^4^

Advances in sequencing technologies have meanwhile allowed huge improvements in detection of small exonic variants.^5 6^ However, despite the gradual transition from exomes to whole genome sequencing (WGS), systematic analysis of non-coding variants has lagged behind. Intronic variants outside the canonical splice donor/acceptor sites can lead to aberrant splicing or creation of pseudoexons; these have been shown to play roles in a range of genetic disorders, sometimes only being identified through RNAseq.^7–9^ Large studies have also shown that strategies including examining constraint of intronic loci and focussing on the putative splicing branchpoint can identify novel pathogenic variants, some of which have subsequently been confirmed by RNA sequencing.^10^ The diagnostic uplift from intronic variants may be as high as 25%^11^ but may differ between disease areas. Improved modelling and machine learning approaches are now also helping to improve screening of intronic variants without the need to perform RNAseq.^12^ Short-read WGS (srWGS) has also been shown to be capable of detecting a very high proportion of SVs, in particular deletions,^13^ with higher accuracy in calling breakpoints than whole exome sequencing. Altogether, cryptic structural and intronic variants may represent a significant source of missing heritability in rare disease.

The 100k Genomes Project (100kGP) was established in 2013 by the UK government and enrolled patients into two distinct arms, cancer and rare disease, between 2015 and 2018.^14^ Approximately 75 000 participants from over 30 000 families were recruited to the rare diseases arm under specific clinical indications (‘normalised disease phenotypes’). Allowing for a discovery-based approach to diagnosis, the project demonstrated the potential of srWGS in diagnosis and clinical decision making and provided genetic diagnoses for 25% of the first 2183 families in the pilot study.^15^ WGS can potentially overcome some of the limitations of standard clinical tests, as in principle it can read across intronic sequences and is capable of determining both balanced and unbalanced SVs, as well as being able to more accurately determine SV breakpoints. These features make it an ideal technique to discover cryptic variants.

One cohort in the rare diseases arm of the 100kGP comprised 56 patients recruited with a suspected autosomal recessive disease in whom only one heterozygous pathogenic variant had been identified by previous clinical testing. WGS performed as part of the 100kGP had already allowed diagnoses to be made in 10 cases in this cohort and among these were cases demonstrating compound heterozygous pathogenic small and SVs. However, cases had been solved on an individual basis and no prior systematic analysis of the cohort had been attempted. With such evidence of compound heterozygous cryptic variants and exonic variants solving cases in this subcohort, we aimed to re-analyse the remaining unsolved cases with a specific focus on cryptic structural and deep intronic variants.

## Methods

Patients were recruited to the 100kGP between 2015 and 2018 from hospitals across the UK. One group of 56 participants was registered under the normalised disease phenotype ‘single autosomal recessive mutation in rare disease’; as previously mentioned, 10 cases had already been solved following analysis via the 100kGP. A further 7 cases were also excluded from analysis due to confirmation of incorrect recruitment to this cohort (recruiting site confirmed no suspicion for a specific autosomal recessive disease) or poor WGS data quality. This left 39 unsolved cases which were suitable for analysis at the time of this study (ie, no second variant yet discovered that accounted for the patient’s phenotype).

The patients presented with a wide range of disorders, ranging from relatively common disorders such as cystic fibrosis to patients without a conclusive diagnosis for complex multisystem disorders. Variant details for 10 cases already solved by the standard clinical ‘TIERING’ pipeline and closed by completion of an ‘Exit Questionnaire’ are shown in online supplemental table S1.

10.1136/jmg-2023-109362.supp1 Supplementary data

WGS was performed on DNA from whole blood by Illumina (HiSeqX, 150 bp paired-end reads). Data for 38/39 unsolved cases were aligned to reference genome GRCh38 with the remaining case aligned to GRCh37 (full list of patients, genes and variants in online supplemental table S2). WGS data from the 100kGP were accessed via the Genomics England Research Environment (GERE), through affiliation with the Oxford Genomic Medicine Centre (GMC). Data release 14 (Main-programme_V14_22-01-27) was used and consent status was confirmed in subsequent data releases. Additional consent to obtain blood samples for validation of the *ENPP1* variant was obtained for P4 under the RUDY study.

**Figure 1:**
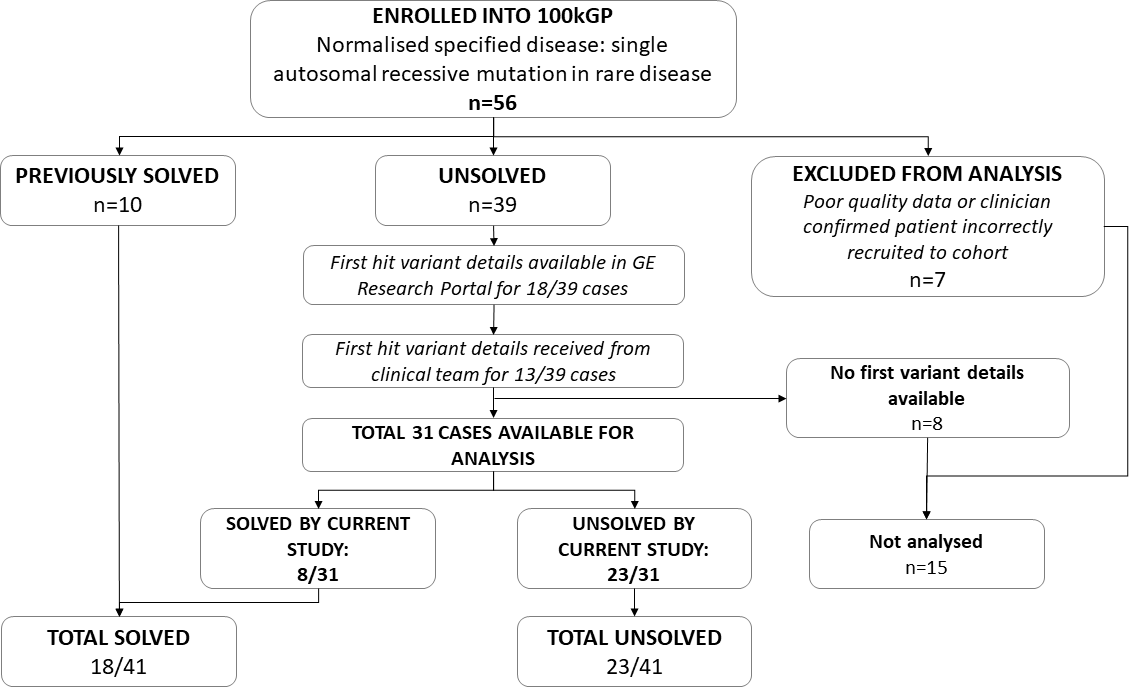
Overall cohort structure. Summary of patients recruited to subcohort ‘single autosomal recessive mutation in rare disease’.

Details of previous genetic testing results (‘first-hit’ genes and variants) were available for 18/39 unsolved participants within the GERE. Clinician contact request forms were submitted for all remaining participants via the research portal Airlock and Genomics England team; details were returned for a further 13 patients. For 8 cases, no information regarding the first-hit variant was available and these could not be analysed further. In summary, information about the first variant was available for 31 unsolved cases (schematic diagram showing study cohort assembly shown in figure 1). Proband data were examined alongside that of family members where available. Presence of the ‘first-hit’ variant was confirmed in all known cases by visualisation in Integrative Genomics Viewer (IGV; V.2.11.9).

Cases were first examined for SVs using SVRare, as previously described.^16^ In brief, SVRare aggregates SV calls made by the Canvas and Manta algorithms and prioritises those found in <1% of all 100kGP participants. An 80% overlap of variants is accepted, so that slight differences in breakpoint calling of the same variant are tolerated. SV calls in or within approximately 1 kb of the same gene as the first hit were assessed. SVRare reports were generated and reviewed for all unsolved participants. BAM and structural VCF files were loaded and assessed visually in IGV (the latter with feature visibility set to zero) for evidence of SVs in the gene of interest. If no pertinent SVs were found, rare small variants were retrieved from the Illumina variant call files using UNIX command line searches and an in-house pipeline that automatically annotates and filters variants. Variants were prioritised based on PHRED-scaled Combined Annotation-Dependent Depletion (CADD) scores and the allele frequency from the 1000 Genomes Project (‘AF1000G’), gnomAD (V.3.1.2) and from the 100kGP.

Further variant characterisation and annotation were performed through database and literature searches. Variants were also assessed for impact on splicing using SpliceAI.^17^ Variants discovered were submitted to the Genomics England Diagnostic Discovery pathway for formal genomic and clinical review by the Genomics England team, according to thresholds agreed by the Diagnostic Discovery Oversight Group. Details of variants meeting clinical thresholds were returned to local Genomic Laboratory Hubs for review and updated reporting, where appropriate.

For P1, the second-hit SV in *CFTR* was validated by PCR and Sanger sequencing. Primers were designed using Primer3Plus^18^ across both insertion breakpoints. Amplification across the proximal breakpoint used oligonucleotides 5’-AGGTTAAGGGTGCATGCTCTT and 5’-TGATTAGAGTATGCACCAGTGGT, while amplification of the distal junction used 5’-TGTCTTGGAGTTGCTCTTCTCG and 5’-AGACATGTGCATGCCAGTCA.

For P4, the SV involving *ENPP1* was resolved by Bionano Optical Genome Mapping using the Saphyr System (Bionano Genomics). Molecules >400 kb in length were retained for analysis (mean fragment length 554 kb) and de novo assembly and variant calling were performed using the Bionano Access software platform. SV calls were compared with a database of 179 individuals from a range of ancestries to exclude common/benign germline SVs. In addition, the variant sequence and breakpoints were confirmed on RNA extracted from blood using nested RT-PCR and Sanger sequencing.

## Results

Putative pathogenic variants were identified in 8/31 of the unsolved families available for analysis. The cases were evenly split between SVs and SNVs, which have been shown to alter splicing. A summary of results for the 8 newly solved cases is given below in table 1. This analysis improved the overall diagnostic yield of srWGS in the cohort from 10/41 correctly recruited cases (24.4%) to 18/41 (43.9%).

**Table 1:** Summary of independent cases solved in this study of unsolved cases recruited to 100kGP due to single autosomal recessive mutation in rare disease

ID	Referral condition	Gene (transcript)	First hit	Second hit (spliceAI/CADD score)	Second-hit type	Comments	References supporting diagnosis
P1	Cystic fibrosis	*CFTR* (NM_000492.4)	c.1521_1523del (p.Phe508del)	Insertion at chr7:117,603,719, NC_000007.14:g.(117603719_117603710)insN(1591) c.2848_2849ins1591 p.(His950Profs*42)	SV (LINE1 insertion)	Partial LINE1 insertions are relatively uncommon in literature. Insertion previously detected 100kGP analysis but interpreted as likely misalignment and discounted.	N/A (novel variant).
P2	Warburg Micro syndrome	*RAB3GAP1* (NM_012233.3)	c.2387_2390del	Deletion of exon 24 and 3’-UTR (chr2:135,165,337–135,511,837) NC_000002.12:g.135165337_135511837del	SV (simple deletion)	Mosaic in father (present in ~44% cells, online supplemental figure S1).	N/A (novel variant).
P3	Pseudoxanthoma elasticum	*ABCC6* (NM_001171.6)	c.2787+1G>T	Deletion of exons 23–29 chr16:16,151,250–16,167,657† NC_000016.10:g.16151250_16167657del	SV (simple deletion)		Common deletion associated with Alu element reported in several studies.^35^
P4	Generalised arterial calcification of infancy	*ENPP1* (NM_006208.3)	N/A (first hit in *ABCC6*‡)	Single complex variant comprised of interlinked duplications of chr6:131837290–131856042 (*ENPP1* exons 2–6) and chr6:129537948–129558439 (*LOC102723409* exons 1–3) NC_000006.12:g.131,856,042_131,856,043ins (NC_000006.12:g.129,537,948_129,558,439inv; NC_000006.12:g.131,837,290_131,856,042)	Single complex SV (two interlinked duplications)	20 Mb ROH region spans both duplicated segments. RNA studies confirm presence of fusion transcript.	N/A (novel variant).
P5	Cystic fibrosis	*CFTR* (NM_000492.4)	c.1521_1523del (p.Phe508del)	c.3874–4522A>G (AG*=0.02/CADD=1.25)	Deep intronic splice variant	Detected due to absence from 1000G Project, not from SpliceAI score. A third unrelated case with same two variants is present in 100kGP but not reported here as was already solved.	Experiments on patient cells and minigene assay results indicate creation of a 125 bp pseudoexon ClinVar VCV000500071.17.^22^
P6	Cystic fibrosis		c.1521_1523del (p.Phe508del)	c.3874–4522A>G (AG*=0.02/CADD=1.25)			
P7	Cystic fibrosis		No variants but *CFTR* in 9.4 Mb ROH region	c.3140–26A>G (homozygous, AG=0.99/CADD=18.5)	Intronic splice variant	Not predicted to affected branchpoint residue (found at adjacent base^36^), but likely causes gain of new splice acceptor site.	Inclusion of 25 bp intronic sequence, creating a frameshift and termination in exon 20, confirmed in RNA studies ClinVar VCV000035864.53.^24^
P8	Jeune syndrome (short-rib thoracic dysplasia 1 with or without polydactyly)	*DYNC2H1* (NM_001377.3)	c.8348A>T p.(Asp2783Val)	c.11049G>A Exon skipping: c.11023_11095del p.(Ile3675Aspfs*2) (AL=0.51)	‘Synonymous’ variant; acts as splice variant leading to exon skipping	First-hit missense variant not present in ClinVar but clinically suspected to be first hit.	Exon skipping causing premature termination in exon 74, confirmed in RNA.^25^

All genomic positions are on GRCh38.

*AG, acceptor gain delta score (SpliceAI).

†Another unrelated case in 100kGP not part of present cohort was solved due to homozygosity of this variant.

‡NM_001171.6:c.1769C>T, p.(Ser590Phe). Variants are heterozygous unless stated otherwise.

AG, acceptor gain; 100kGP, 100k Genomes Project; ROH, region of homozygosity; SV, structural variant; UTR, untranslated region.

### Structural variants

**Figure 2:**
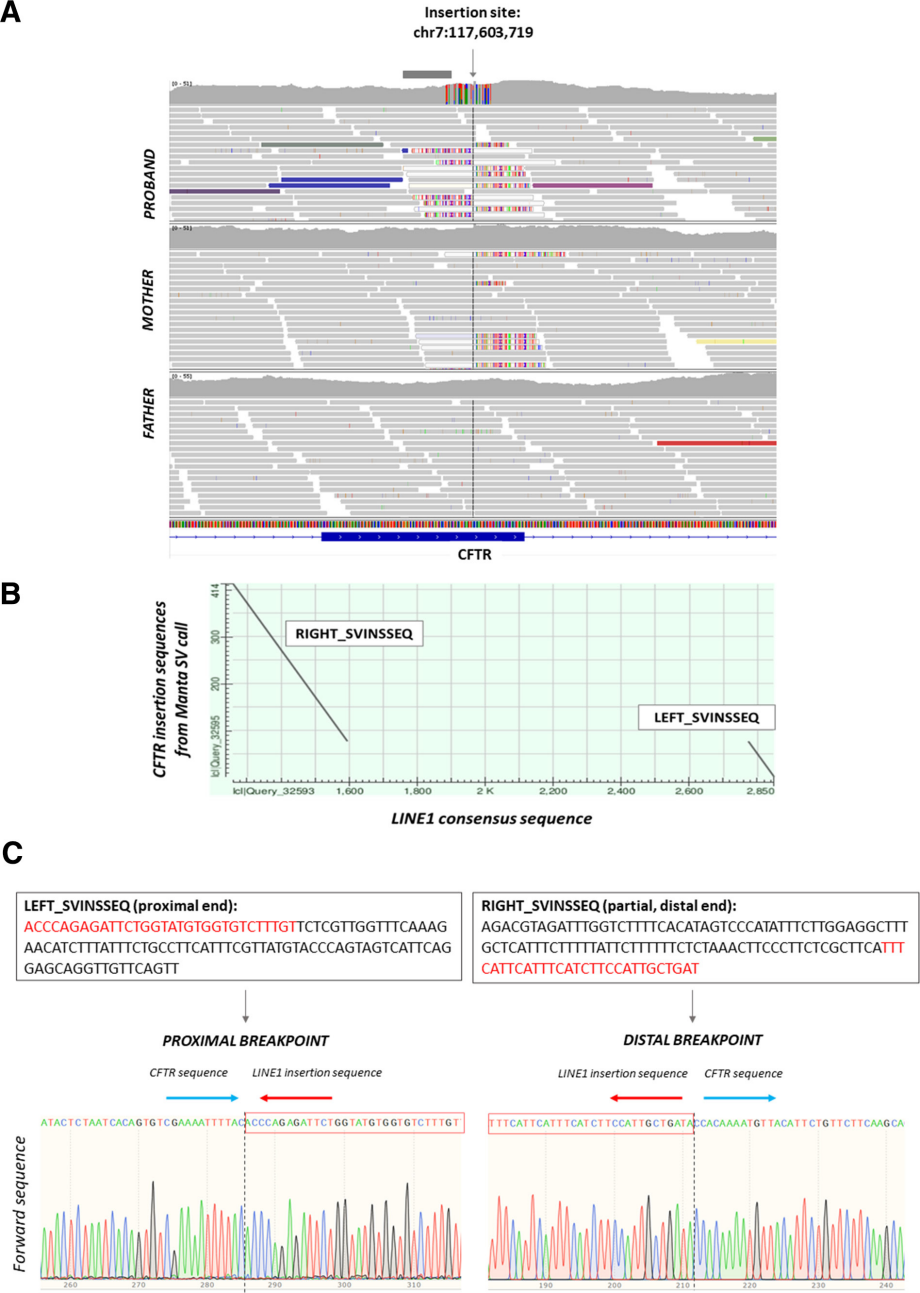
Validation of LINE1 insertion in *CFTR* by Sanger sequencing in P1. (A) Illumina short-read sequencing data supporting LINE1 insertion in P1. Approximate position of MLPA probes (MRC Holland) used during clinical testing is indicated by the grey bar above the coverage track. Left MLPA probe chr7:117,603,631-117,603,659; right MPLA probe chr7:117,603,660-117,603,706. Insertion sequences called by Manta are shown at the proximal (LEFT_INSSEQ) and distal (RIGHT_INSSEQ) breakpoints. Illumina short-read sequencing is unable to read through the full insertion sequence. (B) Dot plot showing comparison of insertion sequences called by Manta to the LINE1 consensus sequence using the BLAST tool at https://blast.ncbi.nlm.nih.gov/. (C) Sanger sequencing to confirm the breakpoint and LINE1 insertion sequence. Sanger sequences using reverse primer (not shown) also confirmed the breakpoint and insertion sequences. MLPA, multiplex ligation probe amplification.

The first patient (P1) presented with classical symptoms of cystic fibrosis including elevated sweat chloride (109 mmol/L) and pancreatic insufficiency. The common northern European founder variant NM_000492.4:c.1521_1523del (p.Phe508del)^19^ had been detected at standard-of-care postnatal screening. Further clinical testing including multiplex ligation probe amplification (MLPA) and a targeted panel (CF oligonucleotide ligation assay kit) that assesses 32 common *CFTR* variants did not detect a second variant; however, analysis of WGS data from the 100kGP subsequently detected an insertion in exon 17 of *CFTR* (figure 2A). Closer examination of the inserted sequence demonstrated mapping to multiple locations in the genome. Although short-read sequencing was unable to span the entire insertion, comparison of insertion sequence to the LINE1 consensus sequence (Human Mispriming Library, https://primer3.ut.ee) showed that the SV was a partial LINE1 element, estimated to be 1591 bp in length (figure 2B) and inserted in reverse orientation into the *CFTR* exonic sequence.

To validate the insertion, primer pairs were designed covering the proximal and distal ends of the insertion. Sanger sequencing confirmed the presence and location of the LINE1 sequence in exon 17 (figure 2C). The inserted LINE1 sequence introduces a stop codon, and the variant was reported clinically using HGVS nomenclature (c.2848_2849ins1591, p.(His950Profs*42)). Since the proband was recruited as a trio with both parents, phasing was confirmed by inheritance, with the p.(Phe508del) variant inherited from the father and the LINE1 insertion inherited from the mother.

**Figure 3:**
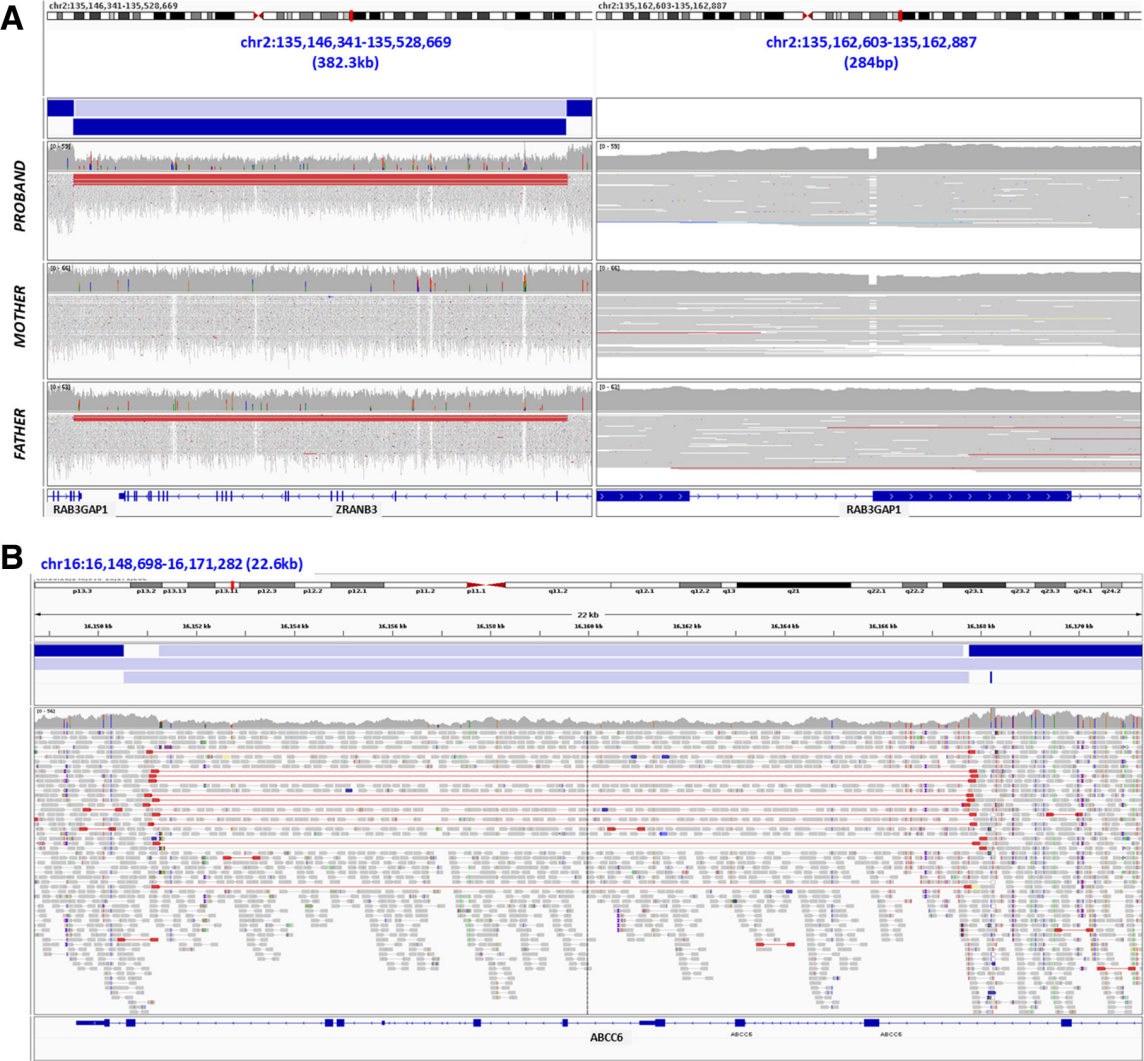
Illumina short-read sequencing data supporting other SVs reported in this study. (A) P2: proband was recruited as part of a trio and data from both parents are also shown. Left panel: deletion of approximately 346.5 kb affecting exon 24 of *RAB3GAP1* and exons 2–21 of *ZRANB3*. The deletion is also present in the father, but is mosaic rather than heterozygous (estimated to be present in 44% of cells (online supplemental figure S1)). Right panel: maternally inherited 4 bp deletion in exon 21 of *RAB3GAP1*. (B) P3: deletion of approximately 16.65 kb affecting exons 23–29 of *ABCC6*. SVs, structural variants.

The second case (P2) had a suspected diagnosis of Warburg Micro syndrome (OMIM #600118) and a 4 bp deletion affecting the splice junction site of *RAB3GAP1* exon 21 was previously detected by Sanger sequencing (NM_012233.3:c.2387_2390del). SVRare detected a deletion of 346.5 kb that affected exon 24 and the 3’-UTR of *RAB3GAP1* (figure 3A), which removes part of the *RAB3GAP1* catalytic domain. Most of the neighbouring gene *ZRANB3* was also removed but no evidence was found to support pathogenicity of this part of the deletion. Phasing by inheritance was confirmed, with the splice variant also detected in the mother, whereas the deletion was present but apparently mosaic in the father. Calculations of coverage in the deleted region versus the wider region gave an estimation of 44% of cells heterozygous for the deletion in the father, with the proband being fully heterozygous (online supplemental figure S1). This would significantly affect recurrence risk estimates, although in this case the parents had completed their family plans by the time of variant discovery, so clinical validation was not deemed necessary.

The third patient (P3) presented with dermatological symptoms of pseudoxanthoma elasticum and visual impairment. A splice donor variant in *ABCC6* intron 21 (NM_001171.6:c.2787+1G>T) was previously detected, which is strongly predicted to affect splicing (SpliceAI DL=0.99) and is reported in ClinVar as pathogenic (VCV000006560.24). SVRare detected a deletion of 17.3 kb involving exons 23–29 of *ABCC6* (figure 3B), which has since been clinically validated by SNP array. This variant would be predicted to cause an in-frame deletion, causing production of a protein without one critical functional ABC transmembrane domain. It has been commonly reported in pseudoxanthoma elasticum and is reportedly mediated by *Alu* elements.^20^ Although the patient was recruited as a singleton and phasing has not been confirmed, this combination of variants is considered highly likely to be causative.

**Figure 4:**
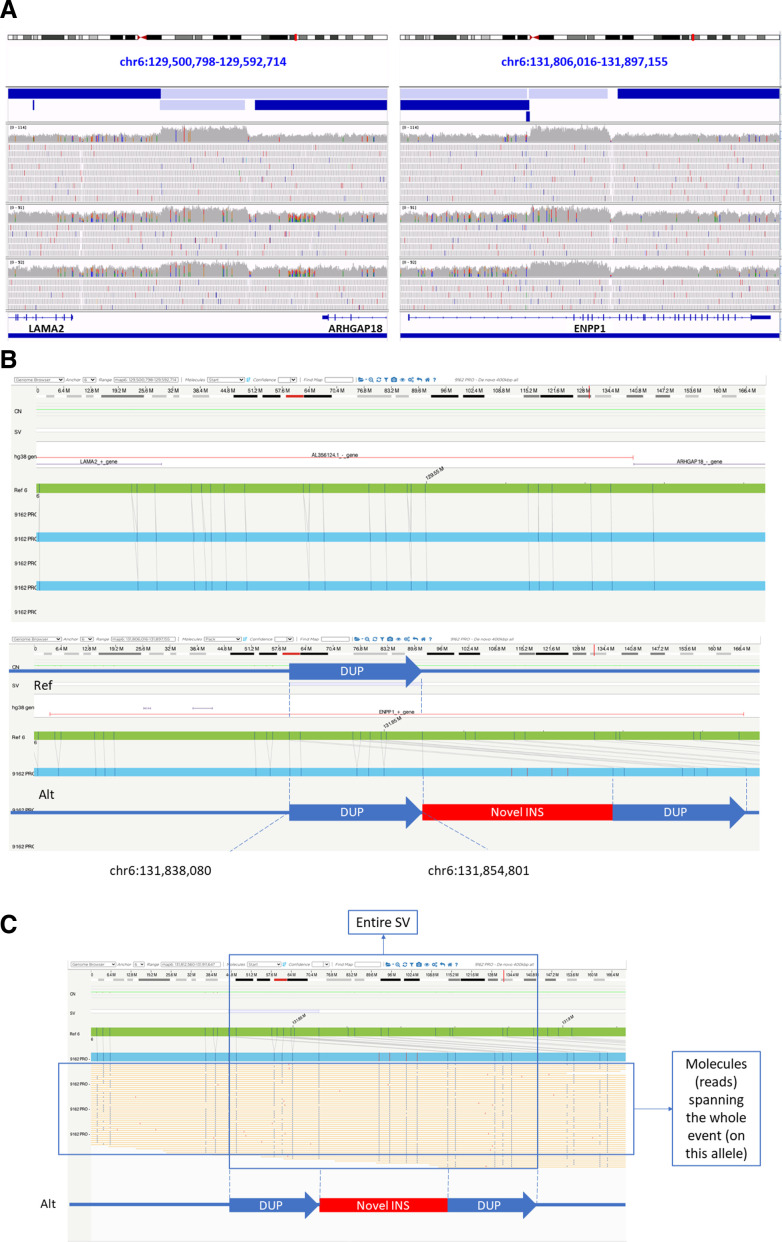
Illumina and Bionano optical genome mapping data supporting *ENPP1* duplications in P4. DNA was extracted, labelled and stained according to Bionano Genomics protocols (Bionano Prep SP Frozen Human Blood DNA Isolation Protocol v2 (Document Number: 30395, Revision: B) and Bionano Prep Direct Label and Stain (DLS) Protocol (Document Number: 30206, Revision: F)). (A) Illumina short-read WGS showing duplications (gain of material) of *ENPP1* exons 2–6 and region between *LAMA2* and *ARHGAP18*. (B) Bionano data showing undisrupted intergenic locus (upper panel) and *ENPP1* duplication (lower panel) with novel insertion (inversion of non-coding *LOC102723409* sequence). Note the *ENPP1* locus mapping to two genomic locations in the lower panel denoted by the vertical and diagonal grey lines spanning from the green reference genome to the blue proband sequence. The diagramatic schema in the lower panel shows the overall interpretation of the SV, with the duplicated region of *ENPP1* sequence separated by the inverted insertion of *LOC102723409* sequence. (C) Single molecule view at *ENPP1* locus showing multiple molecules spanning the entire SV. SV, structural variant.

**Figure 5:**
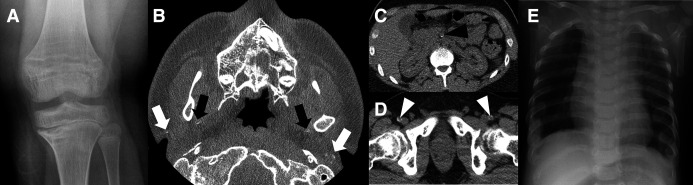
Radiological imaging supporting diagnosis of generalised arterial calcification of infancy in P4 (A–D) and Jeune thoracic dystrophy in P8 (E). (A) Anteroposterior radiograph of left knee aged 12 years demonstrating ‘wide epiphyseal plates of the knee’ with ‘frayed metaphyses of the femora’ and ‘frayed metaphyses of the tibiae’, the latter affecting the medial aspect of the proximal tibia. Appearances are consistent with hypophosphataemic rickets. (B) Axial image from dental CT examination performed aged 20 years. There is mural calcification of the facial arteries (black arrows) and calcification of the included portion of the pinnae of the ears (white arrows). (C) and (D) Axial images from non-contrast urinary tract CT examination aged 20 years. There is mural calcification of the superior mesenteric artery (black arrowhead in C) and both common femoral arteries (white arrowheads in D). Increased density is noted in the renal medullae (C), consistent with early medullary nephrocalcinosis. Calcification of hepatic artery branches was also noted (not shown). In addition, calcification of the popliteal arteries was noted on a CT examination of the lower limbs at age 14 years (not shown). (E) Anteroposterior chest radiograph (aged 6 months) shows a ‘narrow chest’ with ‘short ribs’ and ‘prominent ribs’'. Images annotated using terms from the dREAMS ontology (https://d-reams.org) in inverted commas.

The fourth patient (P4) presented with symptoms consistent with a clinical diagnosis of generalised arterial calcification of infancy (GACI; OMIM #208000), including necrotising enterocolitis supernumerary teeth (case 3 in Merrett *et al* 21) and vitamin D deficiency rickets (online supplemental figure S2A). The patient was entered into the cohort after detection of a missense variant in *ABCC6* (NM_001171.6:c.1769C>T, p.Ser590Phe). MLPA did not identify any copy number variants in *ABCC6,* and no additional variants were detected in *ABCC6* following our analysis of the WGS data. Review of the clinical history revealed *ENPP1* to be another strong candidate gene. Although small variants had previously been ruled out by targeted sequencing, MLPA had not been performed for this gene. SVRare detected a homozygous SV potentially affecting exons 2–6 of *ENPP1*, which would likely explain the patient’s phenotype. This variant lay within a 20 Mb region of homozygosity; the patient was known to be born to consanguineous parents and both parents appeared to be heterozygous carriers (online supplemental figure S2B,C). Close scrutiny of read alignments showed the duplication to be interlinked with a similar sized duplication nearby (table 1 and figure 4A). From the srWGS data, two possible configurations were possible, one of which would not affect the *ENPP1* gene (online supplemental figures S3 and S4A). DNA from peripheral blood from P4 was assessed using optical mapping (Bionano Genomics) to establish the exact configuration of the SV. Disruption of the *ENPP1* locus was confirmed (figure 4B), with duplication of approximately 18.7 kb of *ENPP1* (covering exons 2–6). At each end of the *ENPP1* duplicated sequence, LINE1 (distal) and Alu (proximal) elements were present, leading to difficulty discerning the precise size of the SV. The duplicated *ENPP1* segments were separated by an inverted 23.8 kb sequence. Manual analysis of the labels confirmed that this insertion mapped to a non-coding gene *LOC102723409* situated between *LAMA2* and *ARHGAP18*, located approximately 2.3 Mb upstream of the *ENPP1* locus (online supplemental figure S5). This was therefore consistent with the short-read sequencing data. Nested PCR performed on RNA from blood further confirmed the structure (online supplemental figure S4B,C) and showed a fusion transcript predicted to cause premature truncation of *ENPP1* (p.(Lys239Argfs*9)). Retrospective clinical imaging review also confirmed calcification of joints and wall of the descending aorta, which was consistent with a GACI diagnosis (figure 5A–D).

### Splice variants

Cryptic splice variants were detected in three cystic fibrosis cases in the cohort. These were outside the canonical splice acceptor/donor sites and would likely not have been covered by clinical testing.

The same deep intronic *CFTR* splice variant (NM_000492.4:c.3874–4522A>G) was detected in two cases (P5 and P6). Both patients presented with cystic fibrosis and had been entered into the cohort after detection of the p.(Phe508del) variant. Although reports in ClinVar are conflicting (VCV000500071.24), minigene assays show altered splicing, with inclusion of 125 bp of pseudoexonic sequence, creation of a frameshift and premature transcript termination.^22^ It has also been previously reported in the literature *in trans* with the p.(Phe508del) variant.^23^ Phasing by inheritance was confirmed in one of the cases, with p.(Phe508del) inherited on the maternal allele and the deep intronic variant on the paternal allele. This provides further support for pathogenicity of the intronic variant. Wider examination of the cohort also detected two other patients with the same combination of variants (online supplemental table S1); however, these had already been detected by the GEL pipeline and reported during previous case analysis by the recruiting site.

A near-exonic *CFTR* variant was detected in a fourth patient (P7) with cystic fibrosis. The patient was recruited without a specific first variant detected in *CFTR*, but with a known region of homozygosity covering the whole of *CFTR*. A homozygous splice variant just upstream of exon 20 was detected (NM_000492.4:c.3140–26A>G), which is predicted to strongly affect splicing, with creation of a cryptic splice acceptor site (SpliceAI AG=0.99). Previous RNA studies confirm the inclusion of 25 bp of intronic sequence, creating a frameshift variant, and the variant has been reported >20 times in ClinVar (VCV000035864.54). The variant is associated with a milder phenotype,^24^ including pancreatic sufficiency.

Patient 8 (P8) presented with abnormality of the rib cage (figure 5E) and other features consistent with suspected Jeune syndrome. A missense variant in *DYNC2H1* was detected by WGS (NM_001377.3:c.8348A>T, p.(Asp2783Val)). Although not reported in the ClinVar database, the residue is conserved and is located within a dynein motor loop functional domain. It is also absent from the gnomAD population database and is supported by *in silico* prediction tools (CADD=29.6), so there was strong clinical suspicion of pathogenicity. A synonymous second variant NM_001377.3:c.11049G>A, p.(Pro3683=) was also detected by clinical testing but the significance of this was uncertain at that time. Both variants were seen in the WGS data and *in trans* orientation was also confirmed by inheritance. For the synonymous variant, SpliceAI predicts a moderate impact on splicing (SpliceAI AL=0.51); further literature searches showed the variant has been reported to cause exon 75 skipping and premature termination of the transcript in RNA (p.(Ile3675Aspfs*2)).^25^ It has also been reported *in trans* with a pathogenic *DYNC2H1* exon four deletion in another patient with Jeune syndrome,^26^ providing additional support for pathogenicity using ACMG criteria.

## Discussion

While the 100kGP has provided both new diagnoses and new research insights in rare inherited disease, many patients still lack a genetic diagnosis. Clinical pipelines and tiering strategies such as those used in the 100kGP analysis are inevitably biased towards exonic variants, where there is a much larger body of evidence when annotating potential pathogenicity. Many other studies have also assessed second-hit variants in the 100kGP, but tend to be focused on individual genes or disease phenotypes. This study provides cross-disease strategies to search for cryptic variants in patients with autosomal recessive rare disease where the first-hit variant is known and demonstrates that these variants explain a small but significant proportion of missing heritability. Integration of existing clinical pipelines and research investigations (combined with orthogonal validation for clinical use) may therefore be crucial for obtaining the maximum diagnostic yield from WGS data. This will also help to build evidence supporting the significance of cryptic variants, which will be useful to further refine clinical tiering.

The cases solved in this study were split between structural and intronic variants, indicating that both types of variant are important and can remain undetected in inherited rare disease. It is also plausible that all the second-hit variants found were undetectable by standard-of-care testing, with the exception of P8, where the synonymous variant was detected but not clinically considered as possibly pathogenic. It has been noted that SVs transmitted in the germline tend to be smaller than those arising somatically,^27^ making them more likely to be missed by traditional clinical techniques. For example, in P2 the clinical team that confirmed the resolution of the clinical SNP array design across the relevant region would have been insufficient to detect the variant.

Four of eight solved cases involved patients diagnosed with cystic fibrosis. This is likely due to both the relatively high frequency of the disorder in European populations and the clear, well-established diagnostic characterisations, such that clinical suspicion of the disorder is likely to be correct. Many curated databases and literature reports are also available to aid in variant annotation.

While two solved cases in this project (P2 and P3) were relatively straightforward deletions, P1 and P4 presented more complex solutions. The detection of a LINE1 insertion (P1) is a rare event, and a similar variant has not previously been reported in the literature in *CFTR*. LINE1 insertions in genic regions generally show a bimodal size distribution, with full length insertions (~6 kb) or short fragments (<1 kb) being most common^28^; at ~1.6 kb, this insertion is of intermediate and unusual size. It also was not associated with other features previously reported in retroviral-associated *CFTR* variants: there was no deletion of flanking regions^29^ and the inserted sequence did not contain a poly(A) sequence,^30^ a feature which is suggested to be critical for renewed retrotransposition.^31^ It was also noted that the insertion breakpoint was 2 bp from a AA/TTTT motif known to be the LINE1 endonuclease consensus restriction site.^32^ Across the entire 100kGP, the insertion detected in P1 was private to this family, further confirming its rarity.

Alternative technologies for orthogonal validation will help to improve detection and refine annotation of complex variants. For example, P4 was shown to have a complex variant involving two interlinked duplications. With short-read genome sequencing data, the precise structure was ambiguous. Optical genome mapping using molecules of >80 kb and RT-PCR were therefore critical to prove disruption of *ENPP1*. This provides a more confident diagnosis and allows use in familial cascade testing.

Intronic variants are also chronically underdetected, in part due to lack of annotation and orthogonal validation. All intronic variants in this study had support for pathogenicity from databases or literature, as the small and phenotypically varied cohort would not provide sufficient support for previously unknown variants. The detection of the deep intronic *CFTR* splice variant from P5 and P6 in two previously solved cases (online supplemental table S1) demonstrates a lack of integrated or systematic analysis across this cohort and the wider 100kGP. Interestingly, SpliceAI does not predict any consequence on splicing for this variant (AG=0.02), but functional evidence supports pathogenicity.^22 23^ *In silico* predictions are therefore not always reliable indicators of pathogenicity, highlighting the continued need for orthogonal investigations such as RNAseq. This variant is also demonstrably enriched in cystic fibrosis patients in this cohort. Based on data from GRCh38 only, 15 individuals are heterozygous for this variant across the entire 100kGP (germline data in AggregateV2); of these, 3 (20%) were recruited to this study cohort with symptoms of cystic fibrosis. Overall, the variant is present in 0.02% of patients in the entire cohort compared with 3/11 (27.3%) cystic fibrosis patients in this study cohort.

This was a small cohort, with only 31 cases available for analysis, which limits some of the conclusions made. It is likely that the methods described here could be applied to other patients with autosomal recessive rare diseases and a strong first-hit variant in the 100kGP. However, additional patients may have been recruited under phenotypic categories rather than the specialised cross-disease cohort analysed here, and so are more difficult to find within the larger categories.

Incorrect recruitment was also a confounding factor. As previously stated, 7 cases could be excluded based on feedback from the recruiting sites; inspection of the 10 previously solved cases also demonstrated at least one case with an autosomal dominant condition. Uncertainty over the mode of inheritance and the strength of the first-hit variant may further reduce success in identifying an additional causative variant using these methods. In addition, two cases in this cohort were ultimately solved with homozygous variants. In P7, the region of homozygosity (rather than a specific first-hit variant) along with clear clinical phenotype likely prompted recruitment to this cohort, whereas in P4, the heterozygous first-hit variant at recruitment was misleading. Both homozygous variants were located in wider regions of homozygosity, indicating that such areas should be examined carefully for putative pathogenic variants.

One limitation of this study is that we did not search for variants that impact on distal promoters, enhancers and repressive elements, which may be located up to 1 Mb away from the main promoter of a gene. It has already been demonstrated that SVs can exert effects via regulatory elements, such as in apparently digenic *GJB2/GJB6* non-syndromic hearing loss, where a deletion in GJB6 actually affects a GJB2 regulatory element^33^ approximately 35 kb away. *In silico* predictions and literature database annotation are often lacking for these variant classes, so confidently annotating association and/or pathogenicity in a disorder is difficult. Additionally, the heterogeneous nature of this study cohort meant that we did not attempt to predict variants possibly affecting previously unknown regulatory regions.

Certain genes may also present difficulties due to the presence of segmental duplications or high GC content, both of which can lead to regions of low-quality sequence data. In particular, the proximal part of *ABCC6* (exons 1–9) contains a segmental duplication and the GC content of exon 1 of *GJB2* reaches 80%. It is possible that variants could lie in either of these regions but are not detectable by srWGS due to low mapping and/or sequencing quality scores. This could have affected our ability to detect a second-hit variant in P19 (*ABCC6*) or P30 (*GJB2*).

In conclusion, this study highlights the importance of considering cryptic structural and intronic splicing variants in patients with specific suspected recessive condition where targeted methods have only identified a single heterozygous variant. It also provides strategies and suggestions to complement clinical approaches, demonstrates the importance of collaboration between data analysts and clinical colleagues and reveals the resulting improvement in diagnostic yield. These strategies could therefore improve yield for future WGS analyses when applied to any suspected autosomal recessive disorder, both from the 100kGP and in clinical laboratories. Although the original 100kGP data was limited to short-read sequencing (150 bp paired reads), the use of optical mapping and RT-PCR proved to be critical in resolving the *ENPP1* variant in P4. Selection of the most suitable technology is important, as long-read technologies such as nanopore sequencing typically use shorter molecules than optical mapping, and it might have been more difficult to fully characterise the *ENPP1* variant in P4.^34^ We anticipate that the use of appropriate long-read sequencing technologies and increased knowledge of regulatory elements in the genome will further increase diagnostic yields in similar patient cohorts.

## Data Availability

Data may be obtained from a third party and are not publicly available. 100kGP data are stored in the National Genomic Research Library (https://doi.org/10.6084/m9.figshare.4530893.v6).
