# Supplementary material for: Use of genome sequencing to hunt for cryptic second-hit variants: analysis of 31 cases recruited to the 100 000 Genomes Project
Source: J Med Genet. 2023 Aug 9;60(12):1235–44. doi: 10.1136/jmg-2023-109362 (PMC10715503; doi:10.1136/jmg-2023-109362)
Supplement: Supplementary data [file jmg-2023-109362supp001.pdf]

Use of genome sequencing to hunt for cryptic second-hit variants: analysis of 31 cases recruited to the 100,000 Genomes Project

Supplementary data

TABLE S1:

Genetic variants in 10 cases identified by the exit questionnaire as being already solved at initiation of this study. These cases were therefore not included in the 31 cases reanalysed in this study cohort, as described in the main text and shown in Figure 1.

<table>
<tr><th>Family</th><th>Referral condition (OMIM#)</th><th>Gene</th><th>Variants</th><th>ACMG</th><th>CADD</th><th>SpliceAI_ DS_max & consequence</th><th>Phasing confirmed</th><th>Comments</th></tr>
<tr><td rowspan="2">A</td><td rowspan="2">Muscular dystrophy, limb-girdle, autosomal recessive 1 #253600</td><td rowspan="2">CAPN3 (NM_000070.3)</td><td>chr15:42410446C>T c.2134C>T, p.(Leu712Phe)</td><td>P</td><td>23.9</td><td>0.01 AG</td><td rowspan="2">Yes</td><td rowspan="2">Deletion not called by SV callers; likely due to size (CANVAS) and it is mediated by Alu elements so no clear split reads (Manta).</td></tr>
<tr><td>~32kb deletion CAPN3 exon 1 chr15:42,341,108-42,373,156</td><td>NA</td><td>NA</td><td>NA</td></tr>
<tr><td rowspan="2">B</td><td rowspan="2">Myotonia congenita, recessive #255700</td><td rowspan="2">CLCN1 (NM_000083.3)</td><td>chr7:143331615C>T c.1129C>T, p.(Arg377*)</td><td>P</td><td>38</td><td>0.22 DL</td><td rowspan="2">Yes</td><td rowspan="2">c.1931-24 variant predicted to affect branchpoint of CLCN1 intron 16 (PMID: 30224349)</td></tr>
<tr><td>chr7:143345497A>G c.1931-24A>G, p.?</td><td>LP</td><td>15.22</td><td>0.64 AL</td></tr>
<tr><td rowspan="2">C</td><td rowspan="2">Nemaline myopathy 2, autosomal recessive #256030</td><td rowspan="2">NEB (NM_001164508.2)</td><td>chr2:151524562G>A c.22327C>T, p.(Arg7443*)</td><td>P</td><td>52</td><td>0.01 AL</td><td rowspan="2">Yes</td><td rowspan="2"></td></tr>
<tr><td>chr2:151565752A>C c.18225T>G, p.His6075Gln</td><td>LP</td><td>23.9</td><td>0.98 DG</td></tr>
<tr><td>D</td><td>Intellectual developmental disorder with cardiac defects and dysmorphic facies #618316</td><td>TMEM94 (NM_001351203.2)</td><td>chr17:75491324CAG>C c.1286_1287del, p.Gln429Argfs*28</td><td>P</td><td>31</td><td>0.07 AL</td><td>Yes (heterozygous in both parents)</td><td>In 3.3Mb ROH region but no consanguinity reported</td></tr>
<tr><td rowspan="2">E</td><td rowspan="2">Deafness, autosomal recessive 22 #607039</td><td rowspan="2">OTOA (NM_144672.4)</td><td>chr16:21715058T>G c.1394T>G, p.Met465Arg</td><td>VUS</td><td>24.3</td><td>0.02 DL</td><td rowspan="2">Unclear</td><td rowspan="2">Deletion is part of complex SV and inheritance is unclear</td></tr>
<tr><td>OTOA deletion exons 1-21 chr16:21583121-21736906</td><td>NA</td><td>NA</td><td>NA</td></tr>
</table>

F	Alstrom syndrome #203800	ALMS1 (NM_001378454.1)	chr2:73600799AG>A c.11791del, p.Glu3931Lysfs*18	P	33	0	No	Both coding so 'single hit' may be in a different gene - no information available
			chr2:73448258TA>T c.1732del, p.Arg578Glyfs*17	P	15.65	0		
G	Cerebellar atrophy with seizures and variable developmental delay #618501	CACNA2D2 (NM_001174051.3)	chr3:50376112A>G c.1701+2T>C, p.?	P	33	0.83 DL	Yes (heterozygous in both parents)	In 11.5Mb ROH and consanguinity is reported
H	Achondroplasia #100800	FGFR3 (NM_001163213.2)	chr4:1804392G>A c.1144G>A, p.Gly382Arg	P	23.8	0.05 DG	N/A (autosomal dominant inheritance)	Inherited from affected mother so unclear why this family was submitted under 'Single autosomal recessive mutation in rare disease'. *De novo* VUS in *OFD1* also mentioned in exit questionnaire.
I	Cystic fibrosis #219700	CFTR (NM_000492.4)	chr7:117559590ATCT>A c.1521_1523del, p.(Phe508del)	P	19.21	0.01 AL	Yes	
			chr7-117648320-A-G c.3874-4522A>G	P	1.25	0.02 AG		
J	Cystic fibrosis #219700	CFTR (NM_000492.4)	chr7:117559590ATCT>A c.1521_1523del, p.(Phe508del)	P	19.21	0.01 AL	No	This case was analysed on GRCh37 and so not included in statistics based on AggV2 aggregate file
			chr7-117648320-A-G c.3874-4522A>G	P	1.25	0.02 AG		

TABLE S2: Full breakdown of patients assessed in this study and details of first hit. Cases solved in this study are presented in Table S2a and cases remaining unsolved following this study are presented in Table S2b.

Table S2a: Cases solved in this study

Patient	Variant(s) known at recruitment to 100kGP	ACMG (known variant)	Variant(s) discovered in this project	ACMG (2nd hit)	Phasing confirmed
P1	*CFTR* chr7:117,559,590ATCT>A NM_000492.4:c.1521_1523del, p.(Phe508del)	P	*CFTR* LINE1 insertion chr7:117,603,719	P	Yes
P2	*RAB3GAP1* chr2:135,162,745 TAGAA>T NM_012233.3:c.2387_2390del, p.(Glu796Valfs*12)	P	*RAB3GAP1* deletion chr2:135,165,340-135,511,840 (346.5Kb)	P	Yes
P3	*ABCC6* chr16:16,173,283 C>A NM_001171.6:c.2787+1G>T	P	*ABCC6* deletion chr16:16,151,000-16,167,650 (16.65Kb)	P	No – recruited as singleton
P4	*ABCC6* chr16:16188841G>A NM_001171.6:c.1769C>T (p.Ser590Phe)	VUS (now shown not to be causative)	*ENPP1* interlinked duplications of chr6:131837290-131856042, exons 2-6, (c.241_715dup, p.Lys239Serfs*26) and chr6:129537948-129558439 *Homozygous*	P	Yes
P5	*CFTR* chr7:117,559,590ATCT>A NM_000492.4:c.1521_1523del, p.(Phe508del)	P	*CFTR* chr7:117,648,320A>G NM_000492.4: c.3874-4522A>G	LP	Yes
P6	*CFTR* chr7:117,559,590ATCT>A NM_000492.4:c.1521_1523del, p.(Phe508del)	P	*CFTR* chr7:117,648,320A>G NM_000492.4: c.3874-4522A>G	LP	No – recruited as singleton
P7	CFTR, no variants detected but region of homozygosity noted	N/A	*CFTR* chr7:117,611,555A>G; NM_000492.4:c.3140-26 *Homozygous*	P	No – recruited as singleton
P8	*DYNC2H1* chr11:103,204,858A>T NM_001377.3:c.8348A>T, p.(Asp2783Val)	LP	*DYNC2H1* chr11:103,287,559G>A NM_001377.3:c.11049G>A, p.(Ile3675Aspfs*2)	P	Yes

Table S2b: Cases remaining unsolved following this study

Patient	Variant(s) known at recruitment to 100kGP	ACMG
P9	*LOXHD1* chr18:46,529,227G>A<br>NM_144612.7:c.4480C>T, p.(Arg1494*)<br>*LOXHD1* chr18:46,557,437C>T<br>NM_144612.7:c.3269G>A, p.(Arg1090Gln)<br>*OTOG* chr11:17573104G>A<br>NM_001277269.2:c.2143G>A, p.(Val715Met)<br>*OTOG* chr11:17608400C>T<br>NM_001277269.2:c.4297C>T, p.(Arg1433Trp)<br>*PTPRQ* chr12:80670476A>G<br>NM_001145026.2:c.6586A>G, p.(Met2196Val)<br>*MYO15A* chr17:18,119,911C>A<br>NM_016239.4:c.1111C>A, p.(Pro371Thr)	P<br>VUS<br>VUS<br>VUS<br>VUS<br>VUS
P10	*ETFB* chr19:51,354,305G>A<br>NM_001985.3:c.61C>T, p.(Arg21*)	P
P11	*AGL* chr1:99,884,391A>G<br>NM_000642.3:c.2486A>G, p.(Asn829Ser)	LB*
P12	*CFTR* chr7:117,614,699C>G<br>NM_000492.4:c.3454G>C, p.(Asp1152Met)	VUS
P13	*CFTR* chr7:117,559,590ATCT>A<br>NM_000492.4:c.1521_1523del, p.(Phe508del)	P
P14	*CFTR* chr7:117,559,590ATCT>A<br>NM_000492.4:c.1521_1523del, p.(Phe508del)	P
P15	*CFTR* chr7:117,530,975G>A<br>NM_000492.4:c.350G>A, p.(Arg117His)	P
P16	*CFTR* TG11T5 haplotype<br>NM_000492.4:c.1210-34_1210-6TG[11]T[5]	P
P17	*CFTR* chr7:117,559,590ATCT>A<br>NM_000492.4:c.1521_1523del, p.(Phe508del)	P
P18	*GCM2* chr6:10,877,284G>A<br>NM_004752.4:c.199C>T, p.(Arg67Cys)	VUS
P19	*ABCC6* chr16:16,157,769CA>C<br>NM_001171.6:c.3774CT>C, p.(Trp1259Glyfs*14)	P
P20	*USH2A* chr1:216247094TC>C<br>NM_206933.4:c.2298TG>T, p.(Glu767Serfs*21)	P
P21	*ABCA4* chr1:94031110G>A<br>NM_000350.3:c.4139C>T, p.(Pro1380Leu)	LP
P22	*ATP7B* chr13:51974407G>T<br>NM_000053.4:c.813C>A, p.(Cys271*)	P
P23	*ABCA4* chr1:94011395A>G<br>NM_000350.3:c.5461-10T>C	P
P24	*CEP152* chr15:48,741,986A>G<br>NM_001194998.2:c.3950T>C, p.(Ile1261Thr)	LB*
P25	*PREPL* chr2:44,323,368A>AGT<br>NM_001374276.1:c.1788_1789dup, p.(Leu597Hisfs*4)	P
P26	*ETFDH* chr4:158,685,121G>T<br>NM_004453.4:c.508G>T, p.(Gly123Cys)	VUS
P27	*IGHMBP2* chr11:68,933,864C>A<br>NM_002180.3:c.1488C>A, p.(Cys496*)	P

P28	*ANO5* chr11:22,221,100C>CA<br>NM_213599.3:c.191dup, p.(Asn64Lysfs*15)<br>*ANO5* chr11:22,218,262A>G<br>NM_213599.3:c.155A>G, p.(Asn52Ser)	P<br><br>VUS
P29	*LRP2* chr2:169,182,165 AC>A<br>NM_004525.3:c.9998+1del<br>*LRP2* chr2:169,205,498 A>G<br>NM_004525.3:c.7697T>C, p.(Tyr2566His)	P<br><br>VUS
P30	*GJB2* chr13:20,189,481A>G<br>NM_004004.6:c.101T>C, p.(Met34Thr)<br>8p23.3 deletion also present	P
P31	*NFE2L2* chr2:177,234,075C>T<br>NM_006164.5:c.242G>A, p.(Gly81Asp)	VUS
P32-39	No information available	

**Variants reported by clinical teams as VUS re-classified as Likely Benign using current ACMG/ACGS guidelines. Variant classification tools and criteria may have been updated since recruitment to the 100kGP.*

FIGURE S1:

Figure S1a:

	Proband	Father	Mother
Reads across region of deletion	49875	84800	107787
Reads across chr2 first 10Mb, excluding region of deletion	2797092	3028620	3004141
Ratio of reads in deletion region vs surrounding region	0.018	0.028	0.036
Normalised to unaffected mother	0.497	0.780	1
% decrease in coverage	0.503	0.220	0
% of cells heterozygous for deletion	1.000	0.440	0.000
Predicted status:	***Heterozygous***	***Mosaic***	***Unaffected***

Figure S1b:

rs141436429
7/21 reads
(33%)

rs548515018
9/39 reads
(23%)

rs112900217
7/18 reads
(39%)

rs185932425
5/27 reads
(19%)

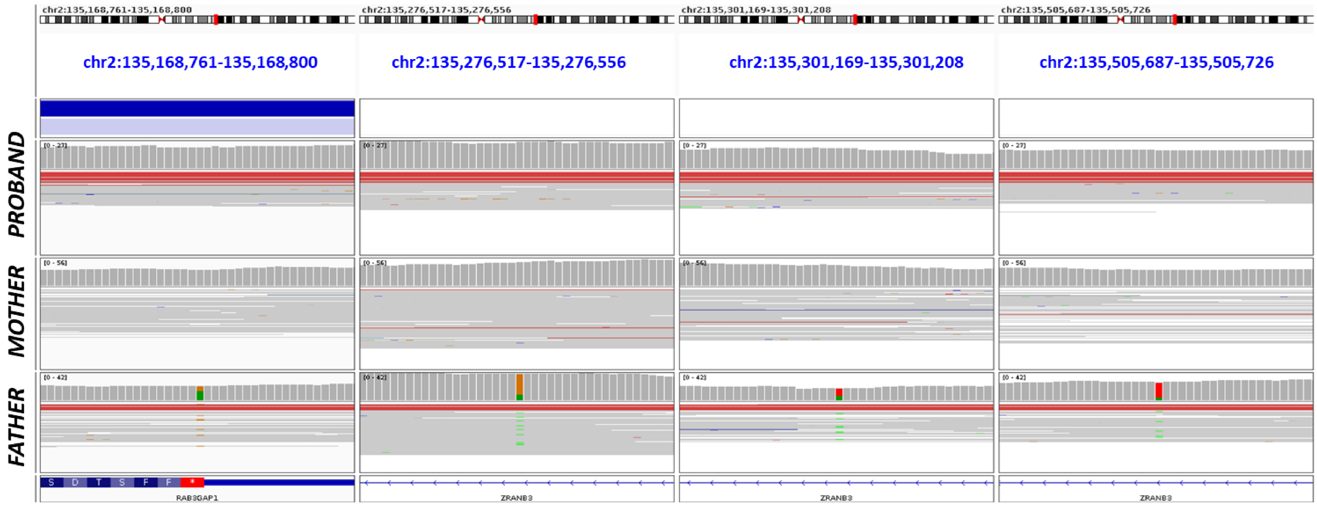

a) Calculation of mosaicism in father of P2, based on difference in read coverage across deleted region between affected and unaffected members of the family.

b) Evidence of mosaicism in father of P2, based on skewing of allele frequency of SNVs. Apparent heterozygosity of SNVs within deleted region confirm the *RAB3GAP1* deletion to be mosaic in the father of P2. IGV screenshot of alignments are shown for all high-confidence heterozygous SNVs (called as PASS and present in dbSNP) within the deleted region. Across these 4 SNVs, the mean allelic fraction of the non-reference allele is 28.5% (range 19-39%). This imbalance translates to an estimate of the deletion being present in 43.1% cells. This is consistent with the read coverage

analysis in Figure S1a, which suggested the deletion to be in 44% cells. Whilst the other 3 SNVs are intronic, rs141436429 is a stop-lost variant NM_012233.3:c.2946A>G (p.Ter982TrpextTer3) listed in ClinVar as a VUS (VCV000436467.17).

FIGURE S2:

a)

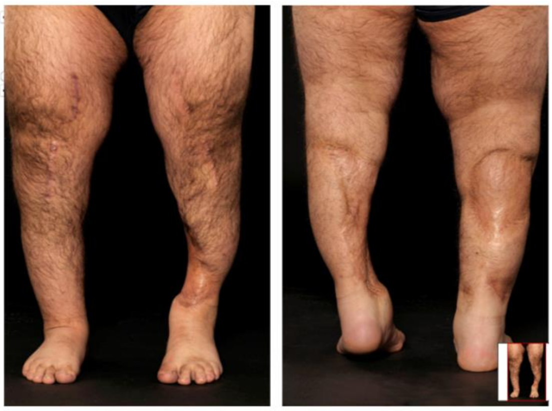

b)

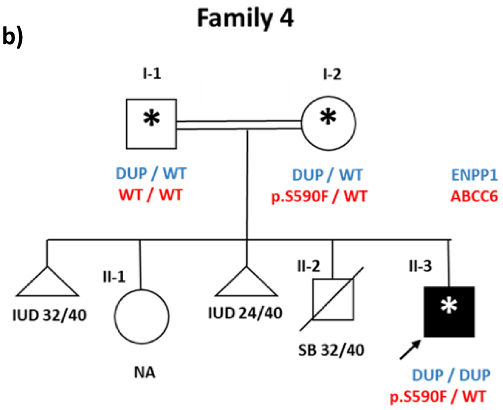

c)

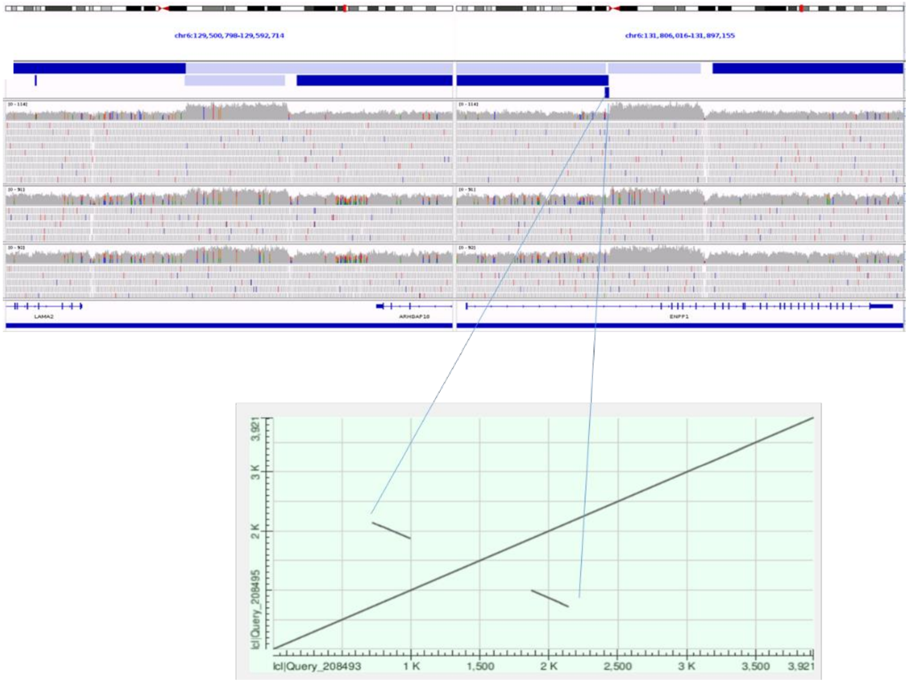

a) Imaging showing rickets in the legs of P4.

b) Pedigree for P4 showing family history of fetal mortality

c) BAM files for P4 and parents visualised in IGV. Duplications in *ENPP1* and in the non-coding *LOC102723409* locus are shown to be heterozygous in both parents and homozygous in the proband.

FIGURE S3:

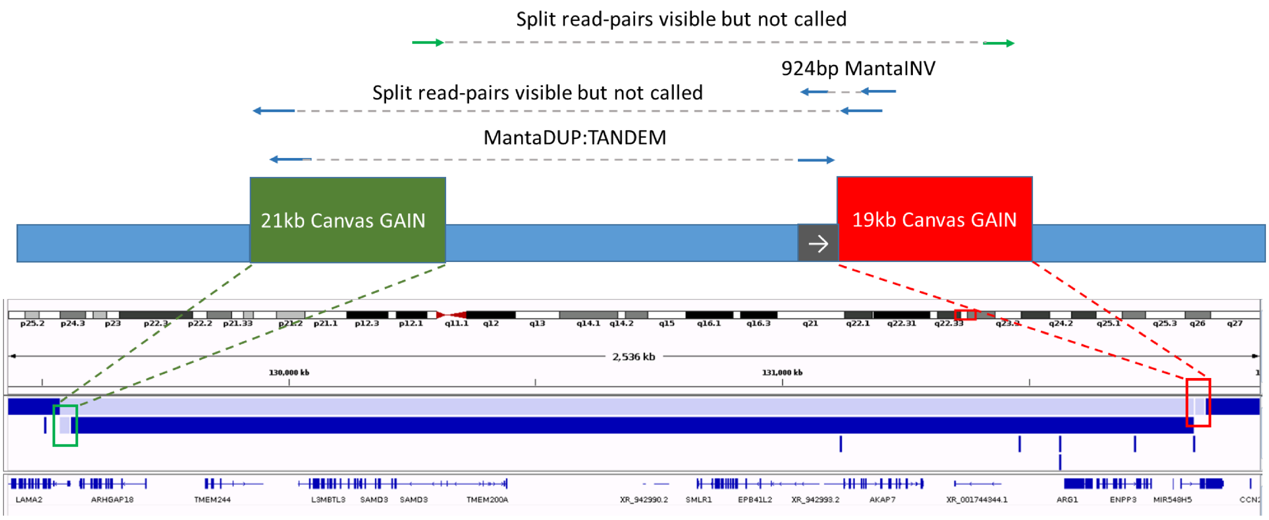

Schematic diagram summarising the SV calls from 150bp paired-end read and the split read-pairs for interlinked duplications on chr6q.

FIGURE S4:

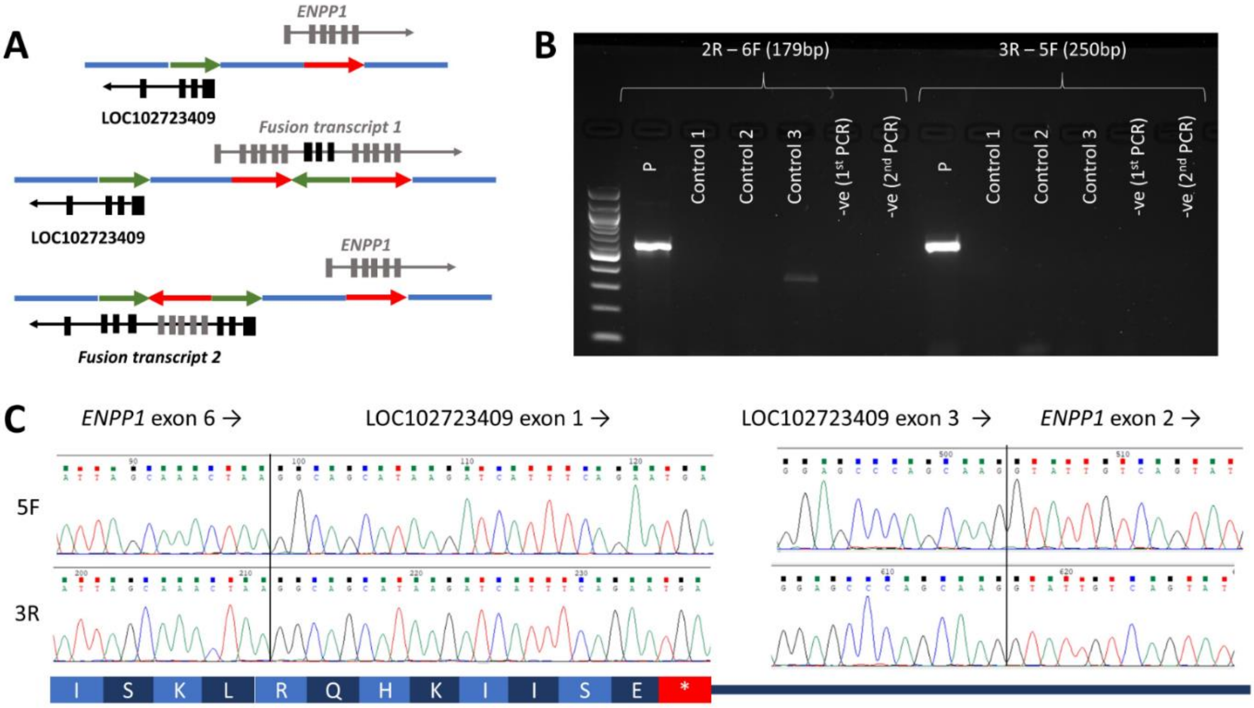

Confirmation of SV configuration and RNA effect using RT-PCR. Peripheral blood was collected into a PAXgene tube and RNA was extracted. RNA quality was confirmed using Bioanalyzer (Agilent, RIN=7.0) and cDNA was generated using the QuantiTect Reverse Transcription Kit (QIAGEN). Nested PCR was performed and products were purified using ExoSAP-IT (ThermoFisher).

a) Schematic diagram showing reference chromosome (top) and two possible SV configurations that would predict two different fusion transcripts. The first configuration (middle) would predict a transcript comprising *ENPP1* exons 1-6, then *LOC102723409* exons 1-3, then *ENPP1* exons 2-end, creating fusion transcript 1 which disrupts *ENPP1*. The second configuration (bottom) would predict a transcript comprising *LOC102723409* exons 1-3, then *ENPP1* exons 2-6, then *LOC102723409* exons 1-end, creating fusion transcript 2 which would leave *ENPP1* intact. Exon numbering of *ENPP1* and *LOC102723409* are based on NM_006208.3 and XR_007059765.1, respectively.

b) Agarose gel image showing RT-PCR results for P4 and two control RNA samples using primers that would only be expected to amplify the fusion transcript configuration 1. Positions of primers relative to the duplicated segment are shown at https://genome.ucsc.edu/s/AlistairP/ENPP1_primers.

c) Bidirectional Sanger data shown for nested RT-PCR using primers 4F&4R then 3R&5F. Similar results were obtained using nested RT-PCR using 3R&5F then 2R&6F. The insertion of exons 1-3 of *LOC102723409* predicts inclusion of 8 novel amino acids (RQHKIISE) followed by a premature stop codon (p.K239Rfs*9). The splice donor site used for exon 1 of *LOC102723409* is within the annotated exon rather than the transcriptional start site.

Figure S5:

Ref sequence (intergenic region between LAMA2 and ARHGAP18)

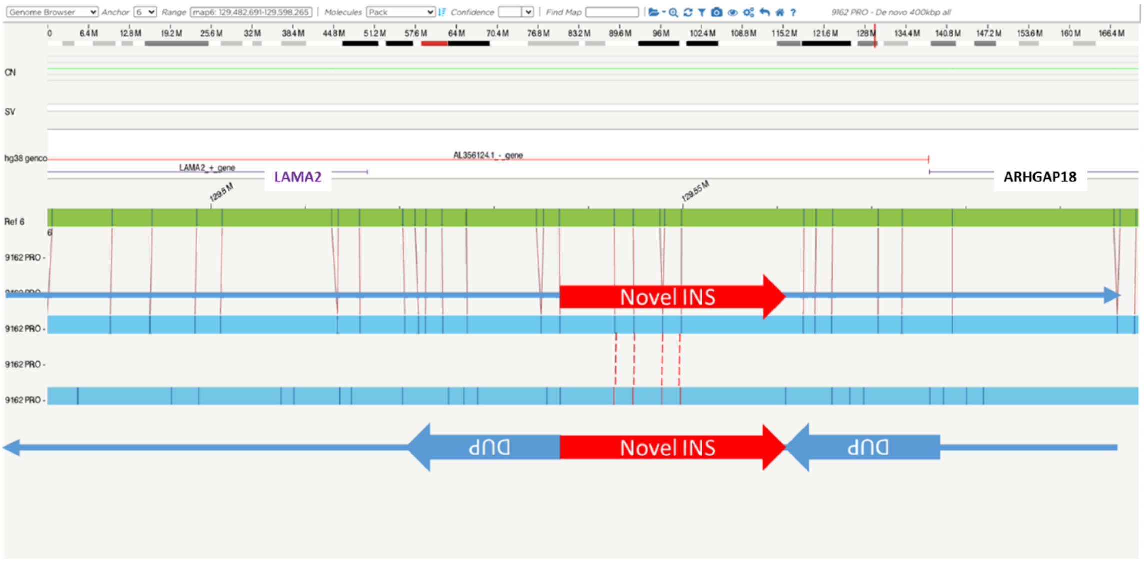

Comparison of label densities indicates that the novel insertion into *ENPP1* perfectly maps to the *LAMA2* locus (when inverted), consistent with fusion transcript 1 shown in Figure S4a.
